# *Drosophila* enhancer-Gal4 lines show ectopic expression during development

**DOI:** 10.1098/rsos.170039

**Published:** 2017-03-29

**Authors:** Sergio Casas-Tintó, Mercedes Arnés, Alberto Ferrús

**Affiliations:** Cajal Institute-CSIC, Ave. Doctor Arce 37, Madrid 28002, Spain

**Keywords:** gene expression, neuroscience, *Drosophila*, development, Gal4

## Abstract

In *Drosophila melanogaster* the most widely used technique to drive gene expression is the binary UAS/Gal4 system. We show here that a set of nervous system specific enhancers (*elav*, D42/*Toll-6*, OK6/*RapGAP1*) display ectopic activity in epithelial tissues during development, which is seldom considered in experimental studies. This ectopic activity is variable, unstable and influenced by the primary sequence of the enhancer and the insertion site in the chromosome. In addition, the ectopic activity is independent of the protein expressed, Gal4, as it is reproduced also with the expression of Gal80. Another enhancer, LN2 from the *sex lethal* (*Sxl*) gene, shows sex-dependent features in its ectopic expression. Feminization of LN2 expressing males does not alter the male specific pattern indicating that the sexual dimorphism of LN2 expression is an intrinsic feature of this enhancer. Other X chromosome enhancers corresponding to genes not related to sex determination do not show sexual dimorphism in their ectopic expressions. Although variable and unstable, the ectopic activation of enhancer-Gal4 lines seems to be regulated in terms of tissue and intensity. To characterize the full domain of expression of enhancer-Gal4 constructs is relevant for the design of transgenic animal models and biotechnology tools, as well as for the correct interpretation of developmental and behavioural studies in which Gal4 lines are used.

## 1. Introduction

The yeast transcription factor Gal4 in combination with artificial gene constructs placed under the control of UAS regulatory sequences became a powerful experimental tool when converted into transgenes in other species [1–3]. In *Drosophila*, this binary UAS/Gal4 system has been extensively used for over two decades [1]. The specificity of the expression domains of Gal4 lines has allowed cellular resolution in most of these studies, representing a major advance over the formerly used genetic mosaics obtained by somatic recombination [4,5]. Beyond the cellular studies, mostly addressing developmental questions, the UAS/Gal4 system has been used also for organismal studies in the fields of neurobiology and behaviour [6–9]. In all cases, however, the space and time specificity of the Gal4 line was the cornerstone of the experiment rationale. Thus, Gal4 lines were described on the bases of their canonical expression domains using a UAS reporter referred to one or several developmental stages. These domains were interpreted as instructions dictated by enhancers located nearby the site of insertion of the Gal4 construct. These lines are generally known as enhancer trap Gal4 lines. In addition, characterized enhancers of a given gene were used to create Gal4 lines with the desired expression domain [10,11]. These lines are referred to as synthetic promoter Gal4 lines.

Enhancers are short (up to 400-bp) DNA sequences that can activate transcription at target promoters located in their vicinity [12–14]. The first transcriptional enhancer was characterized more than 30 years ago, when a viral DNA sequence was shown to activate transcription of the rabbit haemoglobin *beta1* gene, independently from its orientation and position relative to the promoter [15,16]. Eukaryotic chromatin can loop to permit enhancer--promoter interactions in still poorly understood three-dimensional structures [14,17]. Recent genomic studies using various versions of 3C and 4C chromatin immunoprecipitation assays revealed the widespread phenomenon of gene regulation by enhancers while other studies identified specific signatures (histone modifications and associated proteins) of enhancers that greatly facilitate analysis of the databases (reviewed in [18]).

To determine if the expression domains of Gal4 lines are constant along time, and as specific as reported, we analysed several *Drosophila* lines considered to be nervous system specific. Most of them showed transient expression in other tissues. This ectopic activation of enhancers, however, did not necessarily imply the expression of the corresponding genes. Pressure to find utilitarian uses of the limited knowledge on the normal mechanisms of gene expression has led to the production of enhancer-based genetic tools intended to drive expression of engineered genes. However, the range of spurious effects of these applications is rarely analysed in depth, and this justifies further efforts to study how enhancers, both native and synthetic, attain their expression domains.

On the other hand, the temporal analysis of the expression domain of native and synthetic enhancers may help to understand the dynamic process of gene expression along development. Contrary to the widely accepted view that gene expression is a deterministic event, meaning that the final outcome is determined by the initial conditions, our results seem to favour the proposal that, during development, enhancers undergo changing molecular conditions that trigger their variable and ectopic expression. The variability of these conditions is progressively reduced leading to two mutually exclusive events, the extinction of the enhancer's aberrant expression or the consolidation of its canonical expression domain which finally allows transcription of the corresponding native gene and, thus, cell fate determination.

## 2. Results

We selected a collection of *Drosophila* enhancer-Gal4 lines (electronic supplementary material, table S1) described in the literature to be active specifically in the nervous system, to determine if their expression pattern was indeed restricted to the nervous system all along development. For this study we analysed three different scenarios: (i) the activation of enhancers in the original position [E in electronic supplementary material, table S1, *elav^c155^*, D42, OK6, NP2426 *(LN2)*, c105, 796 and *Repo*], (ii) enhancers with their corresponding promoter built into transgenic reporters [P in electronic supplementary material, table S1, *elav (II)*, *elav (III)* and *phantom*]; and (iii) enhancers with a synthetic promoter (P-DSCP in electronic supplementary material, table S1, *GMR10B11* and *GMR 78G09*) (electronic supplementary material, figure S1).

**Figure 1.**
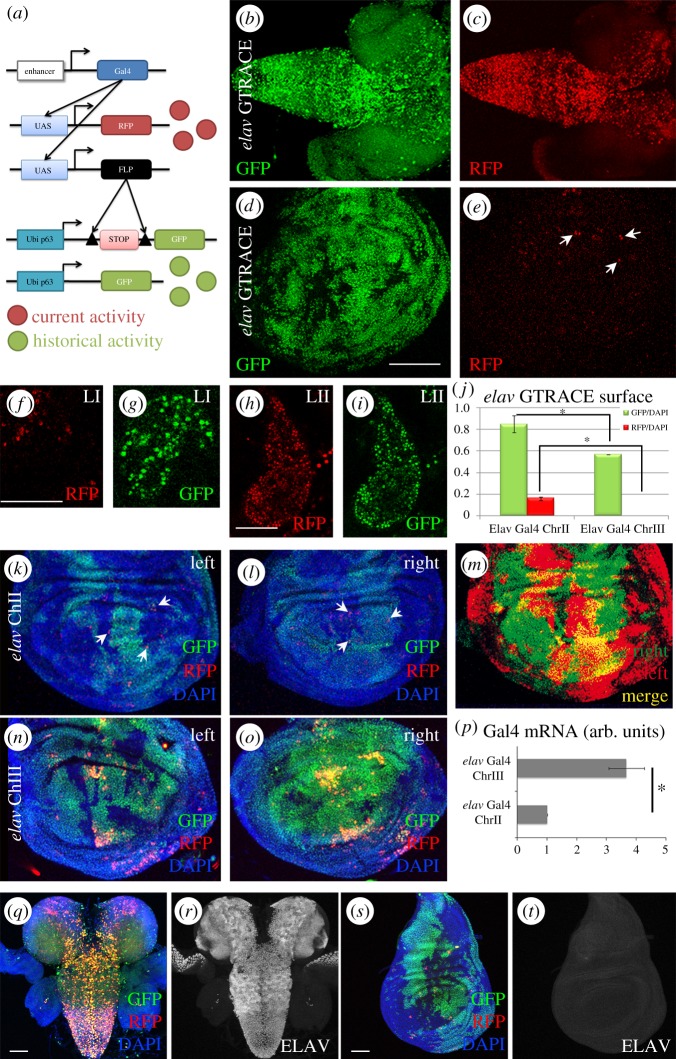
Activation of the neural *elav* enhancer in wing disc cells. (*a*) Schematic description of the G-TRACE technique. An enhancer controls the expression of Gal4 (blue) which results in a red reporter signal (RFP). This red signal will be maintained as long as the enhancer is active, thus, it reflects the current expression domain. On the other hand, the first time in development when the enhancer becomes active the flipase encoding construct is also activated (black box). The flipase, through the excision of a STOP cassette (black triangles), allows the expression of a GFP-encoding construct (green box). This reporter is now controlled by a ubiquitous *p63* promoter, thus becoming independent from the original enhancer. This GFP reporter signal represents the historical expression domain. (*b*–*e*) G-TRACE data from *elav* enhancer in larval brains (*b*,*c*) and wing discs (*d*,*e*). Note that the enhancer is not CNS specific. (*f*–*i*) *In vivo* images of early developmental stages activation of *elav* enhancer in first instar (*f*,*g*) and second instar (*h*,*i*) larvae. (*j*–*o*) Quantification (*j*) of G-TRACE pattern of *elav* enhancer comparing two different *elav*-*Gal4* insertions, chromosome II (*k*–*m*) and chromosome III (*n*,*o*). Note that the ectopic *elav* enhancer expression in the wing disc is not consistent between left and right sides of the same animal. This is evidence of the variable nature of the ectopic expression. (*p*) *Gal4* mRNA quantitative RT-PCRs from chromosome II and chromosome III *elav*-*Gal4* lines. (*q*,*r*) G-TRACE data for *elav* enhancer (*q*) and anti-ELAV staining (*r*) in larval brain. (*s*,*t*) G-TRACE data for *elav* enhancer (*s*) and anti-ELAV staining (*t*) in larval wing imaginal disc. Note that the ELAV protein is CNS specific. Scale bar, 50 µm. Statistics: *t*-test *p < 0,05. *n* = 5 wing discs/sample. Arrows in (*e*) indicate active enhancer cells (RFP).

The G-TRACE technique has been instrumental in this study [19]. Briefly summarized, it consists of three constructs that contain: UAS-*RFP* fluorescent protein, UAS-*Flipase* and Act-FRT-STOP-FRT-*GFP*, respectively. The system reports the temporal activation of the enhancer-Gal4 under study (figure 1*a*, modified from [19]). The enhancer-Gal4 activity induces the expression of the UAS-*Flipase* (Flp) and UAS-*RFP* (red) constructs. The Flp enzyme recognizes FRT sites and removes the STOP cassette, allowing the expression of Act>*GFP* (green) in these cells and their progeny. Thus, we can determine the current expression of an enhancer-Gal4 at the moment of dissection (red, RFP) and its historical expression during development (Act>*GFP* (green)). The inventors of this technique showed already that some enhancer-Gal4 lines exhibit divergence of activity at different stages of development within the same tissue [19].

### 2.1. The nervous system *elav* enhancer is active in epithelial cells

The *Drosophila* gene *elav*, orthologue of human *ELAVL* gene family, is expressed throughout the brain [20–24]. We confirmed the reported *elav*-*Gal4* expression in brain neurons (figure 1*b,c*). The current (red) and historical (green) records of *elav* enhancer activity show a high correspondence between both signals. These data indicate that cells fated to be neurons in the brain are determined during development, and that this fate is maintained during CNS development.

To determine the specificity of this *elav* enhancer, we analysed also epithelial tissues. The data showed unexpected widespread GFP positive cells (figure 1*d*) and randomly distributed RFP positive cells in wing imaginal discs (figure 1*e*) compared to the negative control (sibling flies without the Gal4 construct) (electronic supplementary material, figure S2*a*,*b*). In addition, we analysed the *elav*-*Gal4* enhancer using the Act>STOP>Gal4; UAS-GFP line (see Materials and methods). The data show that *elav* enhancer is active in wing imaginal disc cells (electronic supplementary material, figure S2*c*,*d*) consistent with the previous result. To further validate the initial observation, we used another Gal4 line inserted adjacent to the endogenous *elav-Gal4* enhancer (*elav^c155^*) and the G-TRACE reporter line inserted in chromosome III. In this case, GFP and RFP positive cells were also identified in the wing disc in a pattern similar to the one shown by the previous *elav* construct. Moreover, a detailed analysis of *elav* enhancer activation also showed activity in halter and leg imaginal discs and tracheal cells (electronic supplementary material, figure S2*e*–*l*). Together, these results indicate that the *elav* enhancer, both in foreign or native locations, is active in epithelial cells during development (GFP cells) and maintains its activity by the third larval instar, albeit to a lesser extent [RFP cells are fewer than GFP cells (electronic supplementary material, figure S2*f*–*l*)]. It seems that some wing disc cells switched on this enhancer sometime early in development and still maintained it on by third larval instar (GFP + RFP, yellow cells), while others have switched it off (GFP, green only cells) and others have switched it on lately (RFP, red only cells). Most likely, the RFP signal (red only), occurred several hours before wing discs fixation/dissection and has not had the time to express the FRT/FLP-GFP reporter, a sequential process that takes longer than the RFP reporting (figure 1*a*).

To determine at what stage during development this ectopic enhancer activation occurs, we analysed first and second instar larvae (24 and 48 h after egg laying, AEL) (figure 1*f*–*i*). G-TRACE reporters showed that the *elav* enhancer is active as early as first (figure 1*f*,*g*) and second (figure 1*h*,*i*) instar larvae. Red and green cells are found in the wing disc suggesting that the enhancer activity is triggered at the initial stages of development in this cell system.

To further analyse the temporal activation of *elav* enhancer in this ectopic domain, we used *wg*-*Gal4* (*wingless)* in combination with the G-TRACE system. In second instar larvae, *wg* expression is confined to the wing pouch of the wing imaginal disc (electronic supplementary material, figure S2*m*). However, later during development, this domain is restricted to concentric rings in third instar larval discs (electronic supplementary material, figure S2*n*) [25,26]. These two different expression patterns allowed us to define the temporal activation of the *elav* enhancer. We combined *wg-Gal4*/G-TRACE with a repressor of Gal4 activity, Gal80, under the regulation of *elav* enhancer, aiming to determine the activation of *elav* enhancer by the effect of *elav-Gal80* suppressor on *wg*-*Gal4*/G-TRACE pattern. Our results confirm that the *wg* enhancer is active early during development in wing pouch cells (green in electronic supplementary material, figure S2*o*,*p*) and, at the third instar larvae stage, it is restricted to its canonical *wg* domain (green in electronic supplementary material, figure S2*n* and yellow in electronic supplementary material, figure S2*o*,*p*). Also, *elav*-*Gal80* eliminates the GFP signal corresponding to early *wg*-*Gal4* activity, suggesting that the *elav* enhancer is active during the early stages of development. However, *elav-Gal80* does not suppress RFP signal at third instar stage (electronic supplementary material, figure S2*q*,*r*), confirming that the *elav* enhancer is active during early stages of development in the wing disc.

Next, to discard an effect due to the chromosomal site of insertion, we compared G-TRACE patterns of the same *elav*-*Gal4* construct but inserted in two different sites: chromosome II and chromosome III (electronic supplementary material, table S1). All experiments were carried out in parallel and the results indicate that both *elav*-*Gal4* enhancer insertions have activity during development (GFP cells in figure 1*j*–*o*). However, the insertion in chromosome II is expressed in 60% of the wing cells while the insertion in chromosome III is expressed in 80% of them (figure 1*j*). Moreover, chromosome III insertion has RFP-reported activity in 20% of wing cells (figure 1*j*,*n*,*o*) but activity for the chromosome II insertion is restricted to less than 1% cells (figure 1*j*,*k*,*l*). In summary, the data indicate that even though the *elav* enhancer shows ectopic expression in epithelial cells always, the genomic insertion site affects the extent of this ectopic domain.

We noticed that the expression pattern of GFP throughout different discs is not reproducible in different larvae. This variability could result from heterogeneous environmental conditions among individuals or cell systems. To further evaluate this feature, we compared left and right wing discs from the same individual. Although the number of GFP positive cells is roughly comparable between the two wing discs of the same animal, the pattern differs (figure 1*k*–*o*). This result supports the notion that the enhancer activation during development is not deterministic. We cannot ascertain whether the GFP positive cells activated the *elav* enhancer on a cell autonomous manner, or if they constitute a lineage from one or very few cells that activated the enhancer early in development. However, the fact that the expression pattern is highly variable within the same animal suggests that this is actually a non-clonal event.

Concerning the canonical domain of expression in the CNS, both insertions of the *elav* enhancer show the same pattern in brain cells. However, we addressed possible differences in the intensity of this expression by qRT-PCR. The data show that the expression of the chromosome III insertion is five times higher than that of the chromosome II (figure 1*p*). Together, it seems that the insertion site determines the extent of the ectopic domain as well as the intensity of its expression.

Finally, to determine if the ectopic wing disc expression of the *elav* enhancer induces the expression of the ELAV protein, we stained larval tissues with a specific anti-ELAV antibody. Third instar larval brains show that, as expected, *elav* enhancer is active in neurons (figure 1*q*) and brain cells express ELAV protein (figure 1*r*). However, while wing discs show *elav* enhancer activity (figure 1*s*), we did not detect ELAV protein (figure 1*t*). These results indicate that the ectopic activation of *elav* enhancer does not lead to detectable protein expression.

### 2.2. Toll-6 and OK6 neuronal enhancers are transiently active in epithelial cells in early development

**Figure 2.**
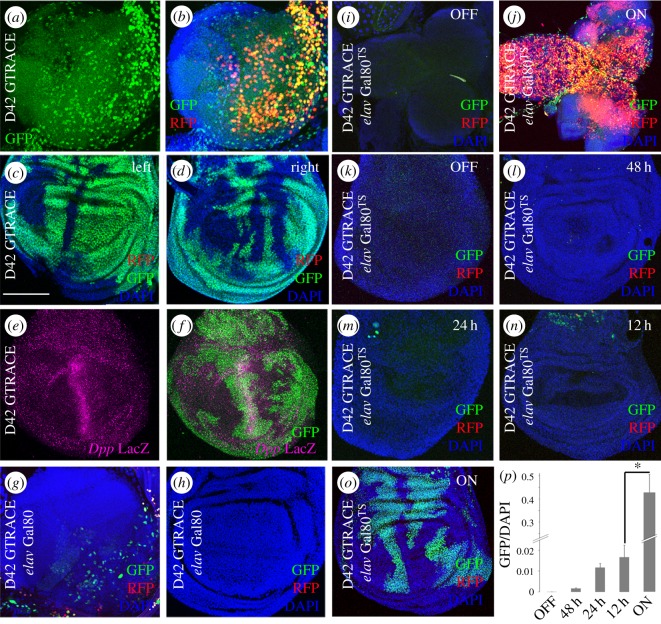
*Toll-6* enhancer in brain and wing cells during development. (*a*–*d*) G-TRACE signal from third instar larval brain (*a*,*b*) and wing disc (*c*,*d*) of the D42-Gal4 line inserted in *Toll-6*. (*e*,*f*) Historical expression of D42 enhancer (GFP) versus LacZ-reported *Dpp* expression (*Dpp*-LacZ, magenta). (*g*,*h*) *elav*-Gal80 suppresses D42-Gal4 activity in the brain (*g*) and wing disc (*h*). (*i*–*o*) Temporal expression experiments in *D42-Gal4>G-TRACE>tub-Gal80^TS^* brain (*i*,*j*) and wing discs (*k*–*o*) maintained at 17°C ‘OFF’ (Gal4 silenced), 48 h, 24 h or 12 h OFF or maintained at 29°C ‘ON’ (Gal4 active). (*p*) Quantification of GFP positive cells per time point. Student's *t*-test *p < 0.05. *n* = 5 wing discs/time point. Bar in (*c*) = 50 µm.

To assess if the phenomenon observed with the *elav* enhancer is general to other enhancers, we selected additional cases also described as CNS specific. Line D42-*Gal4* corresponds to a *Toll-6* gene enhancer [27]. As described, neurons from the third instar larval brain had activated D42 during early development (historical GFP, figure 2*a*, compared to current RFP, figure 2*b*). Similar to *elav*, D42 is also activated in wing disc cells during early development but not in third instar larvae (no RFP cells) (figure 2*c*,*d*). In addition, D42-*Gal4* domain in the wing disc is also different between left and right discs within the same animal (figure 2*c*,*d*). The historical GFP positive cells in the vicinity of the anterior--posterior (A/P) border of the wing disc appear to align with the A/P compartment border. To determine if this is the case, we stained for β-galactosidase in *dpp-LacZ* expressing discs that mark this boundary (figure 2*e*,*f*). The GFP cells distribution does not correspond with the *dpp* enhancer signal.

OK6-Gal4 is another widely used Gal4 line expressed in motor neurons [27] and the Gal4 insertion is located 34 base pairs upstream of the transcription start site of the *RapGAP1* gene (see electronic supplementary material, table S1). We investigated the expression pattern of this driver (electronic supplementary material, figure S3). As reported, OK6 is active in the brain (electronic supplementary material, figure S3*a–c*), but there are GFP positive cells in the wing discs also (electronic supplementary material, figure S3*d*–*f*), revealing earlier activity of this enhancer. Comparing the wing disc expression of the three enhancers, *elav*, D42 and OK6, it is important to realize that the extent of their ectopic expressions is different among them. Thus, ectopic enhancer activity in wing cells is a variable and general phenomenon characteristic of each enhancer and its genomic site.

To discard that the expressions identified by G-TRACE could result from a leaky *Gal4* expression, we repeated some experiments incorporating the Gal4 repressor, Gal80. We combined D42>G-TRACE with *elav*-*Gal80* and analysed the GFP and RFP reporters. Signals in the brain are silenced by *elav*-*Gal80* (figure 2*g* compared to figure 2*a*,*b*). This is a validation of the Gal80 effect on the canonical D42 expression domain in the brain. Next, to validate the inhibitory activity of *elav*-Gal80 during the enhancer ectopic activation, we analysed the wing discs. The ectopic wing expression (figure 2*c*,*d*) is also fully repressed by *elav*-*Gal80* (figure 2*h*). This result confirms that the phenomenon of ectopic expression is not dependent on the protein expressed, Gal4 or Gal80, but it is a property of the enhancer and its environment.

Next, to determine the temporal activation of the D42 enhancer, we used a thermo-sensitive form of the repressor, Gal80^TS^, (electronic supplementary material, figure S4) and analysed the resulting G-TRACE pattern. As controls, we maintained the larvae at 17°C (negative control, figure 2*i*) or at 29°C (positive control, figure 2*j*). The negative control shows no G-TRACE signal and the positive control yields the full enhancer expression in the brain. We set five time points (OFF, 12hOFF, 24hOFF, 48hOFF and ON) corresponding to the hours that the system is kept at the restrictive temperature and, hence, silenced (electronic supplementary material, figure S4). The data show that, if the system is silenced during 48 h, 24 h or 12 h of larval development, the ectopic expression of D42 does not occur (figure 2*k*–*p*). Thus, D42 activation occurs during the first 12 hours of larval development, mainly.

### 2.3. The ectopic activation of enhancers does not lead to expression of their native gene

**Figure 3.**
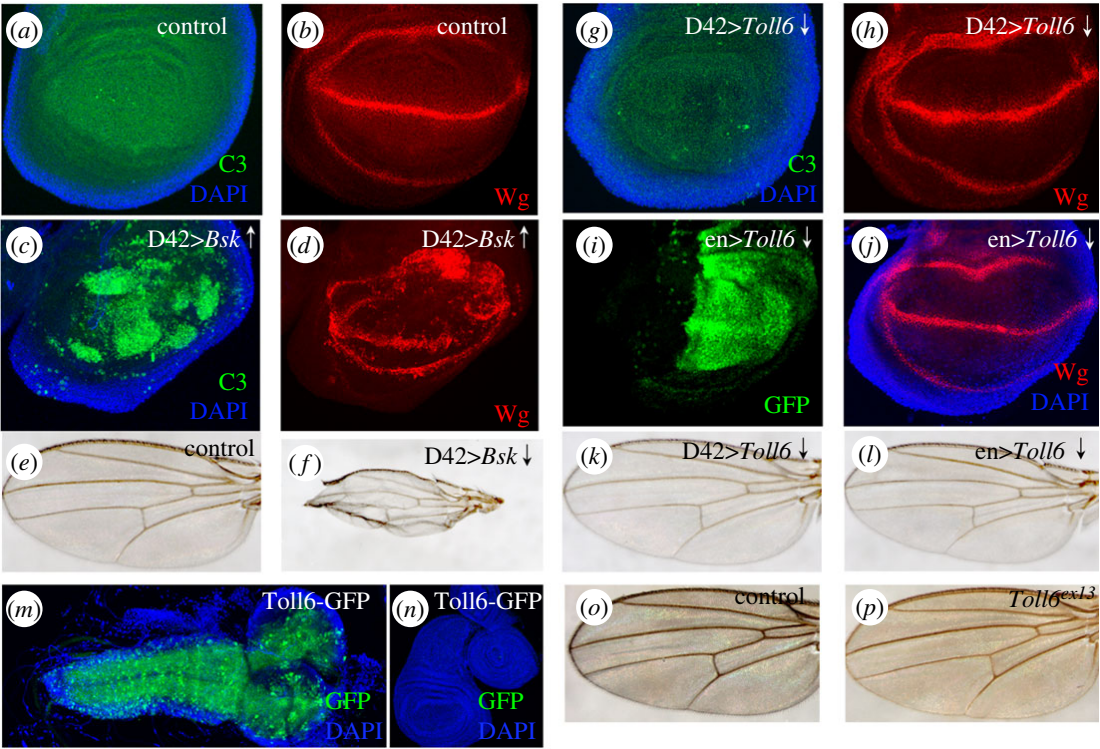
D42 enhancer is expressed in wing cells but its gene *Toll-6* is not. (*a*–*d*) Caspase 3 (C3) (green) and Wingless expression (red) in wild-type (*a*,*b*) and *D42-Gal4>Bsk* (*c*,*d*). Note the extensive apoptosis (*c*) and distorted Wg pattern (*d*) caused by *Bsk*. (*e*,*f*) Resulting adult wings with morphological abnormalities due to the Bsk-elicited apoptosis. (*g*–*j*) Caspase 3 (C3) (green) and Wingless pattern (red) in *D42>Toll-6 RNAi* (*g*,*h*) wing disc. (*i*,*j*) Wingless pattern (red) in en>*Toll-6 RNAi* (GFP) wing disc. (*k*,*l*) Resulting adult wings. Note the lack of morphological effects after inactivating *Toll-6* in the posterior wing. (*m*,*n*) Toll-6-GFP protein trap (Mi{MIC}Toll-6^MI02127^) shows signal in larval brain (*m*) but not in imaginal discs (*n*). (*o*,*p*) Control and *Toll-6^ex16^* mutant adult wings. Cell nuclei are marked in blue (DAPI).

To determine if the ectopic enhancer expression implies a transient expression of the corresponding native gene, we induced apoptosis by driving a core component of the JNK pathway, *Basket (Bsk)*, [28] under D42-*Gal4* control. Wing imaginal discs were stained with anti-caspase 3 (C3) to detect apoptosis, and with anti-Wingless (WG) to monitor wing disc development. Epithelial activation of D42-*Gal4*/*Toll-6* leads to Bsk-dependent apoptosis, as revealed by C3 staining, and to abnormalities in the Wg immune pattern which resulted in defective adult wings (figure 3*a*–*f*). Further, to resolve if *Toll-6* gene expression is necessary for the development of the wing disc, we expressed a *Toll-6* specific RNAi under the control of D42-*Gal4*. We did not detect any apoptosis or morphological defect and the Wg expression pattern was normal (figure 3*g*,*h*). To further demonstrate if *Toll-6* is necessary for wing development, we used an independent *Gal4* (*engrailed, en*) to drive *Toll-6* RNAi expression in the posterior compartment of the wing disc (GFP in figure 3*i*–*l*). The expression of this RNAi with this independent driver did not affect the wing in any noticeable feature. Next, we used a Toll-6-GFP protein trap (Mi{MIC}Toll-6^MI02127^) to detect endogenous Toll-6 protein expression. We observed the canonical Toll-6-GFP signal in the brain (figure 3*m*). However, no signal was detected in imaginal disc (figure 3*n*). These results are in line with reported data showing no detectable *Toll-6* mRNA in wing imaginal discs [29]. To further determine that *Toll-6* expression is dispensable for wing development, we compared wild-type and *Toll-6^ex13^* mutant wings (figure 3*o*,*p*). Mutant wings do not display morphological defects indicating that *Toll-6* expression is not necessary for wing development. Thus, even though the *Toll-6* enhancer D42 is active in the wing disc during development, *Toll-6* is not switched on in the wing, at least to the point of manifesting a visible phenotype in disc size, shape or Wg pattern.

### 2.4. Sequence-dependent enhancer activation

**Figure 4.**
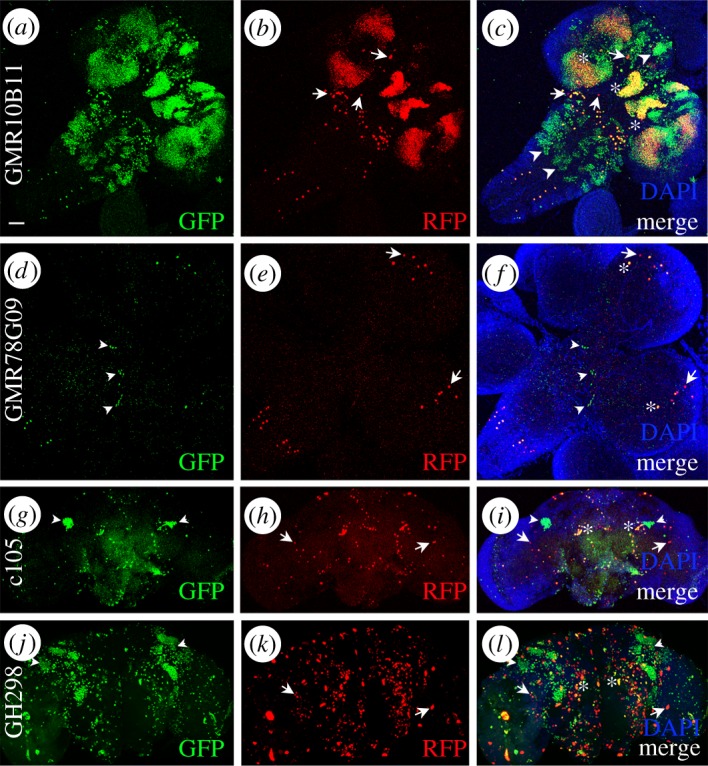
Transient expression of nervous system specific enhancers. GMR10B11-Gal4 (*a*–*c*) and GMR78G09-Gal4 (*d*–*f*) expression revealed by G-TRACE in larval CNS. c105-Gal4 (*g*–*i*) and GH298-Gal4 (*j*–*l*) expression in the adult brain. Note the differences between the historical (GFP) and current (RFP) expression domains in the four enhancers. Since all cells correspond to the CNS, this difference must be considered ‘transient’, rather than ‘ectopic’. Arrows indicate cells with active enhancer (RFP), arrowheads indicate historical enhancer activity (GFP) and asterisks indicate cells with coincident historical and current expressions (yellow). Bar in (*a*) = 50 µm.

We aimed to determine the contribution of the enhancer's sequence to its unstable early activation. To that end, we took advantage of the enhancer-*Gal4* insertions directed to the exact same and insulated chromosomal site with no detectable Gal4 basal activity [30]. Under these conditions, promoters are subject to the same chromatin structural determinants except for the enhancer nucleotide sequence. We compared two same-site/different-sequence insertions: GMR10B11 and GMR78G09 (see electronic supplementary material, table S1). Both insertions are in the 3 L chromosome arm (68A4 polytene band) and show specific activity in the nervous system. G-TRACE experiments confirmed that both enhancer domains are restricted to the nervous system during development. Nevertheless, although GMR10B11 is active in larval brain cells, its historical (GFP) and current (RFP) expressions are not identical (figure 4*a*–*c*). This demonstrates that GMR10B11 expression is transient in certain larval brain cells. GMR78G09 is also expressed in the brain although in fewer cells (about 28 cells, RFP, in third instar larvae). In this case, a group of about 14 cells activated this enhancer in the ventral ganglion during development but not at third instar larvae (figure 4*d*–*f*, arrowheads). These results indicate that the nucleotide sequence of the enhancer is determinant to establish the time and number of cells that will display transient expression during development. In the cases of GMR10B11 and GMR78G09 the non-canonical expression domain is transient but not ectopic since it occurs within the same tissue.

### 2.5. Do all nervous system enhancers have epithelial ectopic activity?

Among the nervous system Gal4 enhancers tested here (electronic supplementary material, table S1) we observed that some enhancers were restricted to the nervous system and showed no activity in wing imaginal disc cells. Enhancers such as GMR78G09 were restricted to a few cells in the larval brain (figure 4*d*–*f*), showing a reduced number of cells with historical expression, which is different from the expression at the moment of dissection (third instar larvae). To verify if some enhancers are less susceptible to ectopic activity than others, we searched for *Gal4* constructs whose activity in the mature adult brain is restricted to the brain. c105-*Gal4* is active in a small number of cells during pupariation and adulthood [8] (see figure 4*g*–*i* and electronic supplementary material, table S1) and GH298-*Gal4* is active in local olfactory interneurons from larva and adult brain [31,32] (see figure 4*j*–*l* and electronic supplementary material, table S1). Our G-TRACE data show that there are some GFP positive cells dispersed in the adult brain and two groups of cells that had transient activity of the enhancer c105 (figure 4*g*–*i*, arrowheads). Also, GH298 was active in clusters of cells along the brain (GFP) (figure 4*j*–*l*, arrowheads). We could not detect ectopic expression in other tissues for these two enhancers.

Further, we searched for domains that are active in a large number of brain cells but with a cellular identity different from neurons. The gene *reverse polarity* (*repo*) is active in glial cells and their precursors during development and throughout adulthood. *repo*-*Gal4* G-TRACE experiments show specific activity in the brain (electronic supplementary material, figure S5) with a full correspondence between historical and actual enhancer activity. Moreover, no ectopic expression could be detected in epithelial wing disc cells. That is, the enhancer *repo* represents a case with neither ectopic nor transient expression. Thus, within the limits of the subset analysed here, all neuronal enhancers display a historical domain which is larger than that of the final stages of development. This extended domain may be ectopic, in most cases, and/or transient. The *repo* enhancer is an exception to this trend. Is this exception marking a difference between neuron and non-neuron cells?

### 2.6. A neurosecretory specific enhancer also shows transient and ectopic activity

**Figure 5.**
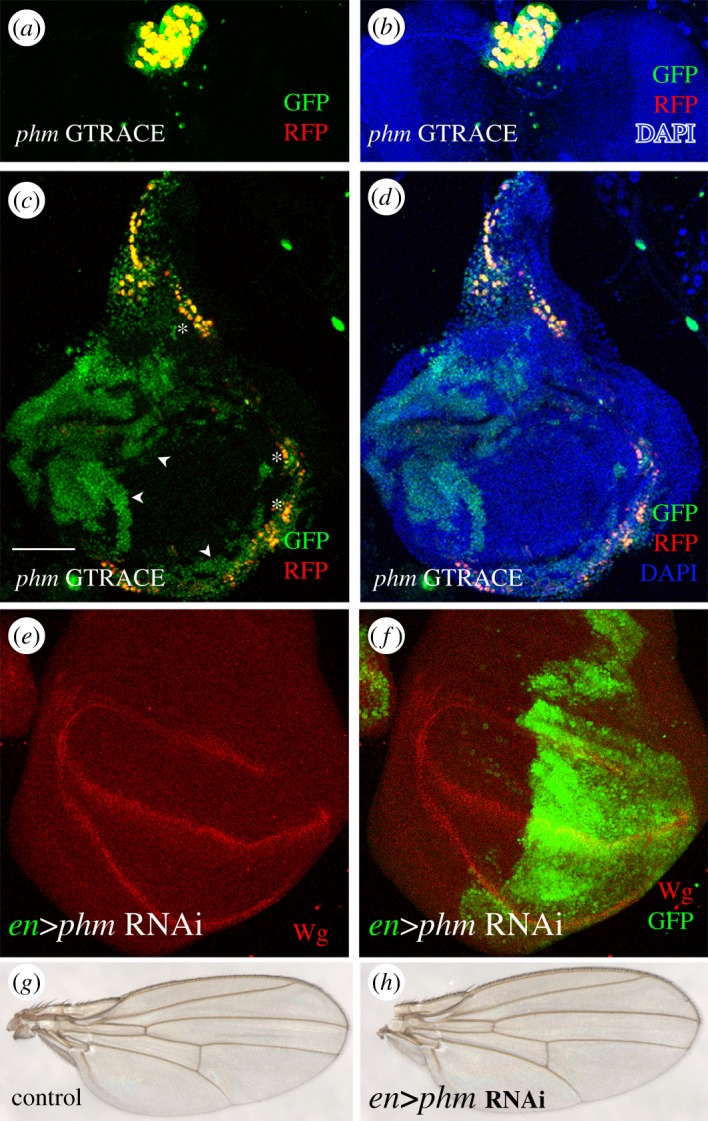
The neurosecretory cells specific *phantom* enhancer. (*a*,*b*) Neurosecretory cells of the larval prothoracic gland expressing the *phantom*-Gal4 enhancer. The expression at the third instar larvae (RFP) is coincident with the historical trace during development (GFP) resulting in all cells yellow. (*c*,*d*) By contrast, the ectopic expression in the wing disc shows a historical trace (GFP) that is larger than the current expression at third instar larvae (RFP). Note cells with GFP only signal (arrow heads) and others with coincident GFP + RFP signal (asterisks). (*e*,*f*) Akin to *Toll-6* (see figure 3), knocking down *phantom* by RNAi in the posterior wing compartment (*en-Gal4>phm-RNAi*) does not alter disc development as judged by the normal expression of Wingless (red). (*g*,*h*) Adult wings of genotypes from (*e*) and (*f*). Note the lack of morphological defects. Bar in (*c*) = 50 µm.

To further analyse neural cells, other than neurons, we studied a case from the *phantom* (*phm*) gene (electronic supplementary material, table S1). This gene is thought to be specifically expressed in neurosecretory cells of the prothoracic gland (PG). However, we recently showed that the *phm-Gal4* driver is expressed in margin cells of the wing disc [33]. The G-TRACE experiments confirm that cells in third instar larvae activate the *phm* enhancer in the PG and in the wing disc (figure 5*a*–*d*). The wing disc also showed historical GFP positive cells at earlier stages of development (figure 5*c*,*d*). Thus, this enhancer of *phantom*, has ectopic activity in epithelial cells early in development.

We have shown above that *Toll-6* expression is not necessary for wing development (figure 3). To investigate this issue in the case of *phm* enhancer, we expressed a *phantom-*RNAi under the control of an independent early enhancer (*engrailed-Gal4*) to knock down *phantom* in the wing disc. Wing disc morphology, size or the expression of the morphogenetic protein Wingless were not affected by the ectopic expression of the *phantom* enhancer (figure 5*e*,*f*). In addition, *phm* knockdown (*en-Gal4>phmRNAi)* does not affect adult wing formation (figure 5*g*,*h*). These data suggest that, similar to *Toll-6*, even though *phantom* enhancer is active in the wing disc, *phantom* gene expression is not required for wing cell development.

### 2.7. Sexual dimorphism in the transient enhancer expression

**Figure 6.**
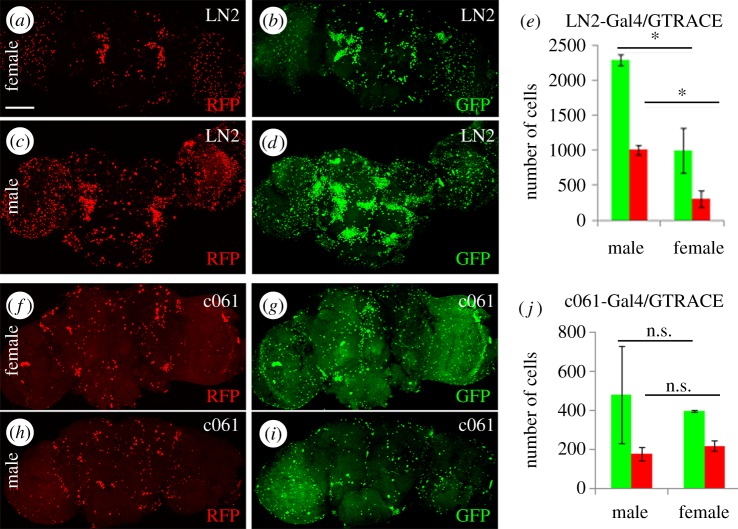
Sex differences in enhancer expression domains. G-TRACE analysis of 4-day-old adult brains expressing LN2-Gal4 (*a*–*e*) or c061-Gal4 (*f*–*j*). Female (*a*,*b*) and male (*c*,*d*) adult brains express the LN2 enhancer during development (GFP) and in its current pattern (RFP). (*e*) Quantification of LN2 active cells. (*f*–*i*) c061-Gal4 expression pattern. Female (*f*,*g*) and male (*h*,*i*) adult brains show activity during development (GFP) and in adulthood (RFP) but, contrary to the case of LN2, there is no evidence of sex differences in the extent of the expression domain. (*j*) Quantification of c061 data. Statistics: Student's *t*-test *p < 0.05. Number of brains/sample = 5. Bar in (*a*) = 50 µm.

In our study, we included the LN2-*Gal4* line that is inserted in the 5′region of the gene *sex-lethal (Sxl)* aiming to explore the behaviour of an enhancer in the context of sex. LN2-*Gal4* is expressed in a set of olfactory local and projection neurons that arise from the lateral neuroblasts [34]. We carried out G-TRACE experiments to verify that LN2-*Gal4* expression is restricted to the adult brain and we did not find early expression during embryonic or larval stages. In the adult brain, we found expression concentrated in two groups of neurons symmetrically distributed; presumably olfactory interneurons as it was previously described (figure 6*a*, arrows). Some additional neurons also activate LN2-*Gal4*, particularly in the optic lobes (figure 6*a*). However, when the historical expression of LN2-*Gal4* was analysed, the number of neurons with earlier expression was noticeably more extensive than the current expression domain (figure 6*b*,*d*,*e*). Since LN2 was not expressed during embryonic or larval stages, these supernumerary neurons must have activated the enhancer either during metamorphosis or during the initial hours of adulthood. Thus, similar to GMR78G09 and GMR10B11 above, LN2 shows transient, rather than ectopic, expression.

Further analysis revealed that males and females show differences in the activity of this enhancer. Males display more LN2 expressing cells per brain than females (figure 6*c*,*d*,*e*) and the transcription of Gal4 in LN2-*Gal4* is stronger in males than in females as shown by PCR assays (electronic supplementary material, figure S6*h*). Since the *Sxl* gene is located in the sexually dimorphic X chromosome, we analysed if this effect could result from chromosomal dosage compensation. To that end, we tested another neural Gal4 line inserted in the X chromosome, c061. This line is expressed in the fan-shaped body, dorsal protocerebrum, mushroom body and dopaminergic neurons [35–37]. The data from adult brains showed a significant difference between the historical and current expression domains, the former being more extensive than the latter in line with all other enhancers of this study (figure 6*f*–*j*). However, no sex differences were detected in the expression domains of c061 (figure 6*j*) or in the transcription levels of *Gal4* (electronic supplementary material, figure S6). To further validate these results, two additional X-chromosome neural enhancers were tested, *796*-*Gal4* and *elav^c155^*-*Gal4*. These lines are P-element insertions in the endogenous *ccb* [38] and *elav* [39] genes, respectively (electronic supplementary material, table S1). *796*-*Gal4* flies were crossed by UAS-*RFP* to mark cell membranes and UAS-*synaptobrevin-GFP*, to visualize synaptic zones. No sex dimorphism was observed in the volumes of either of these cellular domains (electronic supplementary material, figure S6*a*–*e*). In addition, we compared *elav^c155^-Gal4*/G-TRACE male and female ectopic expression in wing imaginal discs (electronic supplementary material, figure S2*h*). No sex differences were detected either in the historical (green) or in the current (red) ectopic activation pattern of this enhancer (electronic supplementary material, figure S2*f*,*g*).

To clarify if the sexual dimorphism observed in LN2-*Gal4* was determined by the sex of the cell or by the peculiar nature of this enhancer, we feminized these cells by co-expressing a construct from the *transformer* gene, *UAS-tra^F^* [40]. In this genotype, the feminized males still show the high number of LN2-Gal4 expressing neurons as regular males do (electronic supplementary material, figure S6*i*,*j*). Thus, the differential expression of LN2 is an intrinsic feature of this enhancer rather than an indirect consequence of the sex-determining function of *Sxl*.

## 3. Discussion

This study has revealed that enhancers may be activated in ectopic domains early in development. This non-canonical expression is always transient although it can be sustained until the end of the last larval stage. However, we have not found evidence that the transient expression of an enhancer leads to the transient expression of the corresponding gene. The type and extent of the ectopic expression depend on the enhancer sequence, as well as on the genomic localization site.

In spite of the unstable expression pattern at early stages of development, all enhancers analysed in this study eventually consolidate into a canonical expression domain that corresponds to the nervous system. Within the enhancer set analysed here, ectopic domains include preferentially the imaginal discs (see electronic supplementary material, figure S2*f*–*i*). Whether this feature is related to the common ectodermal origin of neural and epithelial tissues is an issue worth analysing in the future. Other tissues were routinely screened during the dissection but found mostly negative. Within the nervous system, some enhancers show ectopic expression meaning that the historical trace of activity spans more neural cells than the final expression domain. We refer to these cases as transient domains to differentiate them from the bona fide ectopic expression meaning a different tissue. Transient and ectopic, however, may be considered two variants of the same phenomenon that differs from the canonical expression.

As enhancers modulate transcriptional activity of promoters, it is important to emphasize that the phenomenon of transient and/or ectopic enhancer activation is reproduced with different promoters. In particular, the study of *elav* was performed with three different transgenic lines. The P-element inserted in *elav* locus (*elav^c155^*) accounts for native enhancers and promoter. By contrast, *elav-Gal4* transgenic lines inserted in chromosome II and III contain the native sequence of *elav* enhancer plus a minimal promoter from the P-element. Despite these differences, in the three cases ectopic activation occurred.

Enhancer activity is determined by chromatin structures and, ultimately, by the binding of protein complexes to DNA. The *Sex lethal* enhancer LN2 exhibits sexual dimorphism in its ectopic expression domain. LN2 is expressed in more cells in males than in females, both in the ectopic and in the canonical expression domains. All other enhancers analysed do not seem to show sexual dimorphism. *Sex lethal* is known to be regulated by splicing mechanisms that determine transcript isoform expression according to sex [41,42]. Thus, the sex-dependent regulation of LN2 identified here suggests that a different mechanism must operate on the activation of this enhancer with respect to that of other enhancers. This alternative mechanism is independent of the sex identity of the cell and it is regulated by intrinsic characteristics of the enhancer because the feminization of male cells did not alter the sexual dimorphism in its expression. It is plausible that this feature might reflect sex differences in chromatin structure at the LN2 locus or in the repertoire of binding transcription factors involved in sex determination. The speculations proposed here for the LN2 enhancer of *Sxl* would be akin to the genomic specializations described for general sex determination in plants and animals including *Drosophila* [43–45]. In any case, it seems that the mechanisms that underlie unstable enhancer expression are diverse but not unspecific.

The ectopic expression identified through the G-TRACE system is not artefactual because it is effective to trigger the subsequent expression of the coupled gene, illustrated here with *basket*. Thus, we can conclude that the observations provided by the G-TRACE technique reflect the normal course of events during development. That accepted, the question arises of what type of filter the genome uses to extinguish the unstable expression to finally consolidate a canonical expression domain. Since ectopic domains may consist of single isolated cells, to relatively large cohorts of adjacent cells, extinction by signalling from neighbouring cells seems unlikely. For an autonomous cell, perhaps chromatin locus specific mechanism seems more likely. The case of sex-dependent LN2 enhancer may indicate that the activation and extinction mechanisms are site specific. We hypothesize that the process of enhancer activation follows a sequential history in which the early steps are largely variable and refinement into the canonical domain is progressively built. The molecular microenvironment at the enhancer site, rather than enhancer sequence, affects the early steps of this process. However, the final expression domain is determined by the enhancer sequence. The two examples of the *elav* enhancer analysed here support this conclusion. It is worth noting that the initial steps of this process, although variable, cannot be considered random throughout the organism. The ectopic wing expression domains of *elav*, for example, although variable among and within larvae, always affect the wing disc and not the fat body, for instance. Thus, it is likely that the first step in the enhancer activation process may be constrained already to some extent. This study leaves open, however, the issue of the mechanism to identify and extinguish the ectopic expression of enhancers. The pillar of evolutionary change is variability and subsequent selection. Thus, we can envision that the unstable activation of enhancers, although regulated to some extent, could be modified by any number of factors (e.g. hybridization, heterochromatin rearrangements, viral infections, environmental clues, etc.) in a way that would escape the filter mechanism for expression extinction and, thus, eventually result in a change of the canonical expression domain of a gen.

The initial instability in enhancer expression may be relevant to properly evaluate the use of genetic engineering in biotechnology and the interpretation of a plethora of developmental biology studies where Gal4 lines are used. It seems appropriate to carry out extensive analyses on the historical expression of enhancers before launching studies based on the utilitarian use of gen constructs in the, so-called, selective expression domains.

## 4. Material and methods

### 4.1. *Drosophila* genetics

All fly stocks were maintained at 25°C (unless otherwise specified) on a 12/12 h light/dark cycle at constant humidity in standard medium. The following stocks were used: *elav*-Gal4^c155^ (BL#458), *elav*-*Gal4* (BL#8765), *elav*-*Gal4* (BL#8760), *D42-Gal4* (BL#8816), *repo-Gal4* (BL#7415), *GH298-Gal4* (BL#37294), c105 (BL#30822), *c061-Gal4* (BL#30845), *GMR10B11-Gal4* (BL#48247), *GMR78G09-Gal4* (BL#40015), G-TRACE (BL#28280), G-TRACE (BL#28281), *UAS-bsk* (BL#9310), *Tubulin-Gal80^TS^* (BL#7019), *tub-GAL80^TS^* (BL#7019) *Toll-6^ex13^* (BL#64072), *Toll-6-GFP* Mi{MIC}Toll-6^MI02127^ (BL# 34467), *P{Act5C-Gal4}* (BL#3954) and (BL#42713), *UAS-Tra.F* (BL#4590) are from Bloomington Drosophila Stock Center. Line *LN2-Gal4* (NP2426-Gal4, Kyoto#104198) is from Kyoto Stock center, *phantom*-*Gal4* is a gift from M.B. O'Connor, *elav*-*Gal80* is a gift from G. Morata, *796-Gal4* [38] was generated in our group and *OK6-Gal4* [27] is a gift from C. O'Kane.

### 4.2. Immunostaining

Third instar larvae and adult brains were dissected and fixed with 4% formaldehyde in phosphate-buffered saline for 20 minutes, washed three times with 0.1% triton, and mounted in Vectashield mounting medium with DAPI, or incubated with primary antibodies anti-active-caspase 3 (1/100, Cell Signaling), anti β-gal (1/50, DSHB), anti-elav (1/50, DSHB) or anti-Wingless (1/20, DSHB), and secondary antibodies Alexa 568 or 647 (Life Technologies). Preparations were imaged by confocal microscopy with Leica SP5 microscope. Fluorescence quantification was performed with Imaris software. Images were processed with ImageJ.

### 4.3. Statistical analysis

Statistical significance was calculated using a Student's two-tailed *t*-test, with significant differences between compared groups noted by *p < 0.05.

### 4.4. Live imaging

First and second instar larval wing imaginal discs were visualized and imaged following a previously published protocol [46].

### 4.5. Quantitative RT-PCR

Total RNA was isolated from 15 fly heads per genotype (Trizol, Invitrogen); cDNAs were synthesized with M-MLV RT (Invitrogen). Gal4 Taqman probe (Sc04172924_s1) and RNA-pol II (housekeeping gene) Taqman probe (Dm02134593) were used (Applied Biosystems). qPCR analysis was done using 7500 Real Time PCR System (Applied Biosystems) with cycling conditions of 95°C for 10 min and 40 cycles of 95°C for 15 s and 55°C for 1 min. qPCR results were analysed with 7500 v.2.0.6 software (Applied Biosystems).

### 4.6. Statement on data and reagent availability

All the strains and reagents are available in the repositories indicated in Materials and methods section. Electronic supplementary material, table S1 contains all the Gal4 lines tested.

## Supplementary Material

Supplementary Figure S1

## Supplementary Material

Supplementary Figure S2

## Supplementary Material

Supplementary Figure S3

## Supplementary Material

Supplementary Figure S4

## Supplementary Material

Supplementary Figure S5

## Supplementary Material

Supplementary Figure S6

## Supplementary Material

Supplementary Figure S7
