# Supplementary figures and images for: Drosophila enhancer-Gal4 lines show ectopic expression during development
Source: R Soc Open Sci. 2017 Mar 29;4(3):170039. doi: 10.1098/rsos.170039 (PMC5383858; doi:10.1098/rsos.170039)

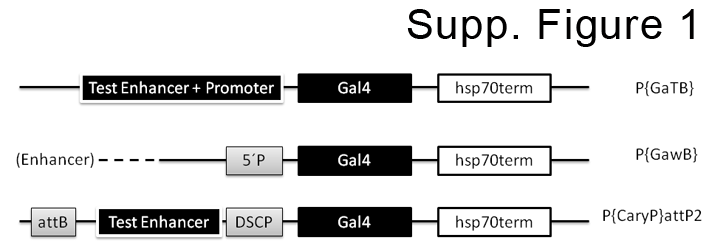

Supplement: Supplementary Figure S1 [file rsos170039supp1.tif]

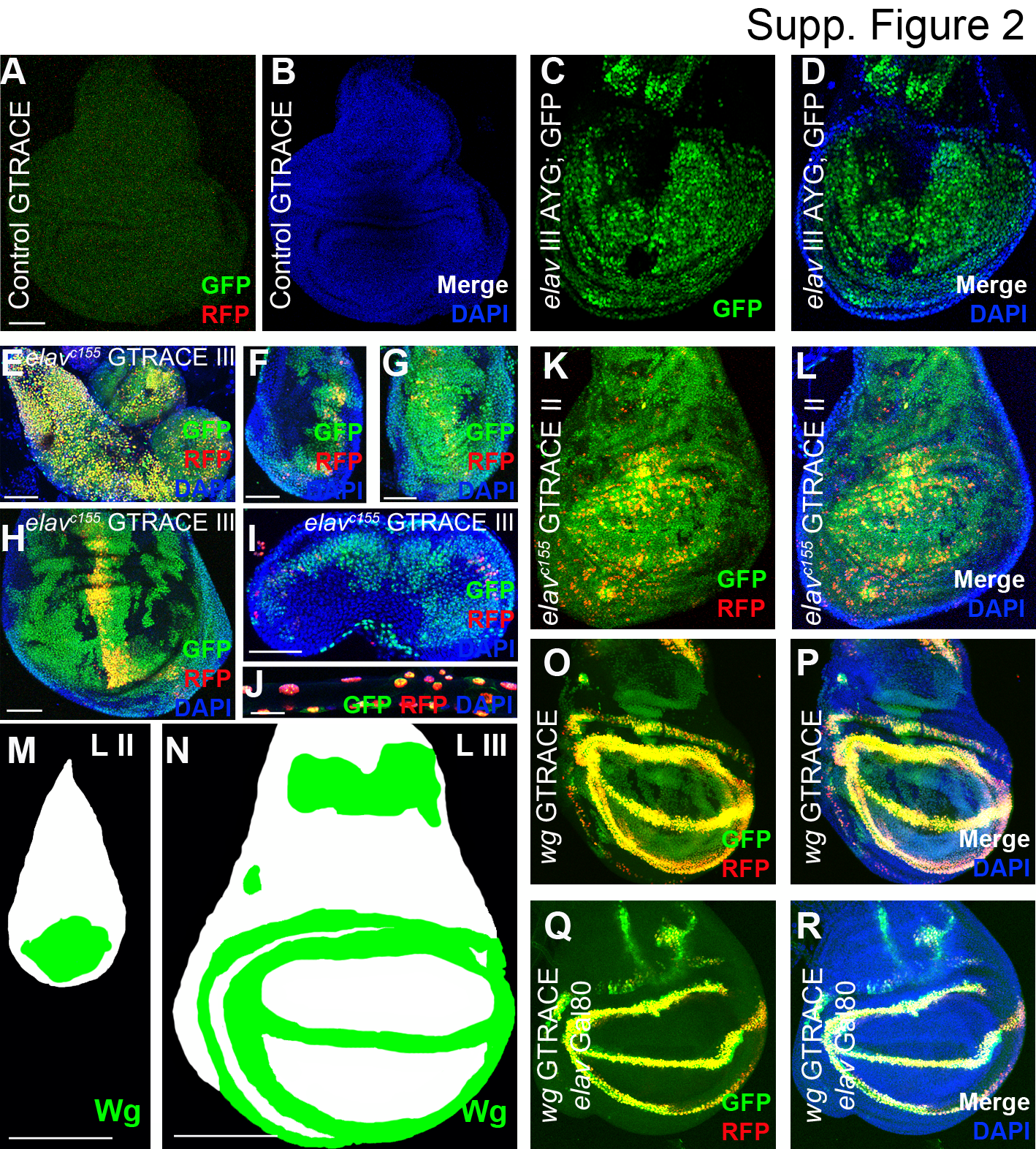

Supplement: Supplementary Figure S2 [file rsos170039supp2.tif]

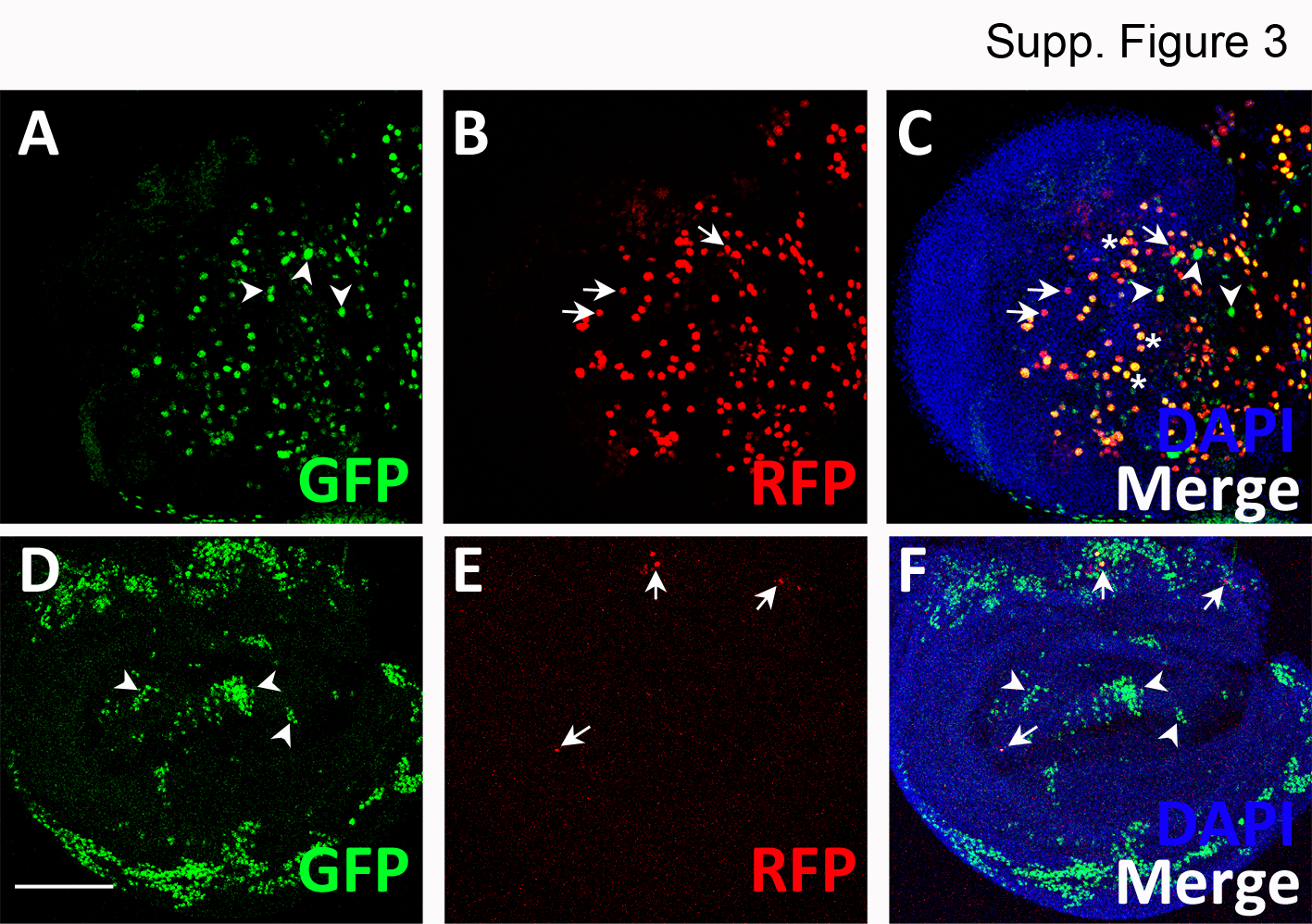

Supplement: Supplementary Figure S3 [file rsos170039supp3.tif]

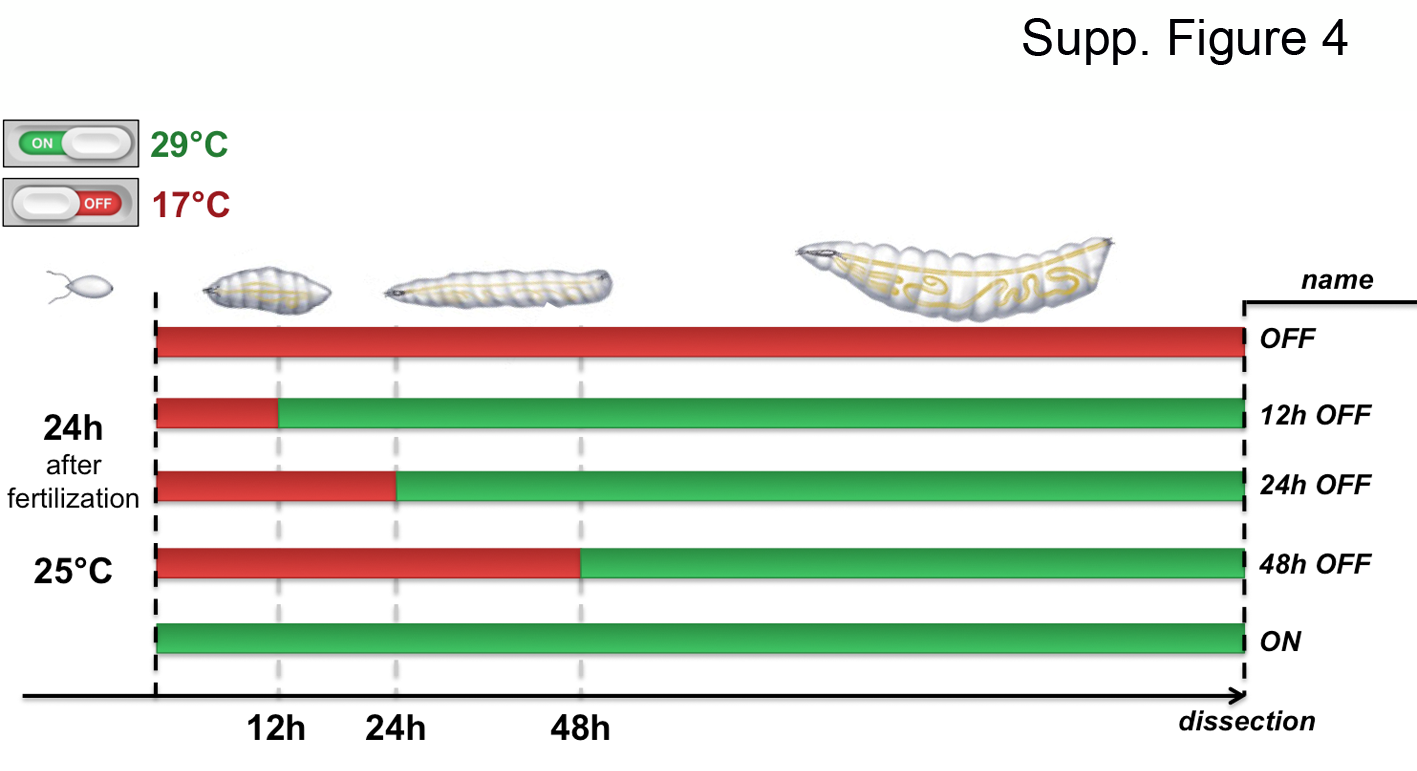

Supplement: Supplementary Figure S4 [file rsos170039supp4.tif]

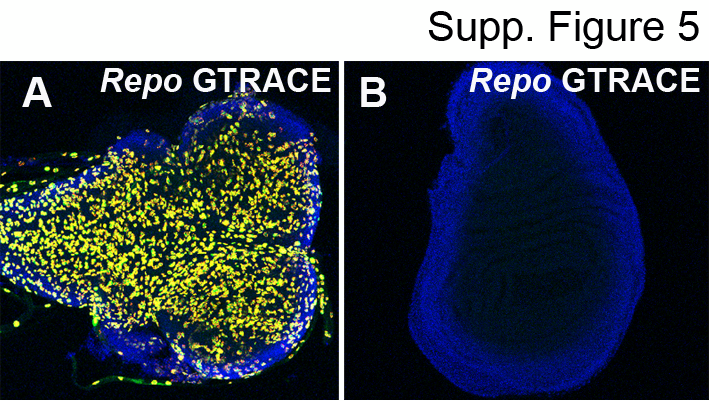

Supplement: Supplementary Figure S5 [file rsos170039supp5.tif]

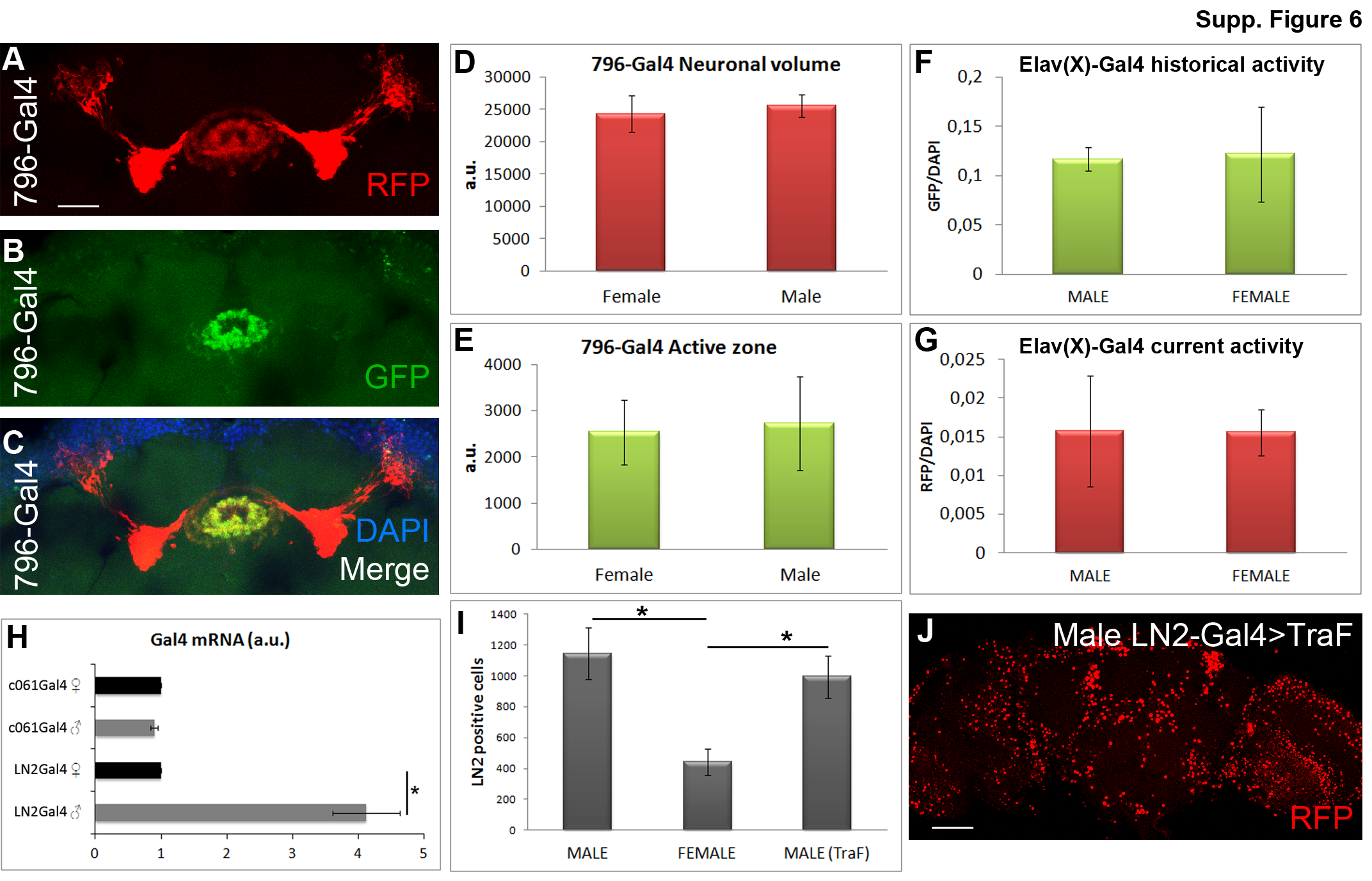

Supplement: Supplementary Figure S6 [file rsos170039supp6.tif]
