# Supplementary material for: Drosophila enhancer-Gal4 lines show ectopic expression during development
Source: R Soc Open Sci. 2017 Mar 29;4(3):170039. doi: 10.1098/rsos.170039 (PMC5383858; doi:10.1098/rsos.170039)
Supplement: Supplementary Figure S7 [file rsos170039supp7.docx]

| **Gal4 driver** | **Gene** | **CNS** | **Wing disc**  **Early** | **Wing disc**  **L3** | **Sequence location** | **Bloom. Ref.** | **Construct** | **Reference** |
| --- | --- | --- | --- | --- | --- | --- | --- | --- |
| elav^c155^  (Chr.X) | *Elav* | +++ | +++ | +++ | X  (1B8):523,350..523,350 [-] | 458 | E | *Lin et al., 1994* |
| elav (Chr.II) | *Elav* | +++ | ++ | ++ | 2 | 8765 | P | *Luo et al., 1994* |
| elav (Chr.III) | *Elav* | +++ | +++ | +++ | 3 | 8760 | P | *Luo et al., 1994* |
| D42 | *Toll6*  7075 bp upstream of the  transcription start site | +++ | +++ | No | 3L (71C2):15,329,625..15,329,625 | 8816 | E | *Chan et al., 2002* |
| OK6 | *RapGAP1*  34 bp upstream of the transcription start site | ++ | ++ | + | 2L (28B1):7,576,638..7,576,638 | 64199 | E | *Babcock et al., 2004* |
| NP2426  (LN2) | *Sex lethal* | ++ | No | No | X (6F5):7,096,129..7,096,129 [-] | 104198 (Kyoto) | E | *Das et al., 2008 & Seki et al., 2010* |
| Phantom | *Phantom* | No | + | ++ | 3 |  | P | *Ono et al., 2006* |
| C061 | unknown | ++ | No | No | X | 30845 | - | *Krashes et al. 2009* |
| C105 | *βNACtes6*  insertion 49 bp upstream of the transcription start | + | No | No | X (12E8):14,317,167..14,317,167 [+] | 30822 | E | *Renn et al., 1999* |
| GMR  10B11 | *Inositol 1,4,5,-tris-phosphate receptor* | + | No | No | 3L (68A4):11070538..11070538 | 48247 | P-DSCP | *Jenett et al., 2012* |
| GMR  78G09 | *Sex peptide receptor* | + | No | No | 3L (68A4):11070538..11070538 | 40015 | P-DSCP | *Jenett et al., 2012* |
| Repo | *Reversed polarity* | +++ | No | No | 3R  (90F9-90F10) | 7415 | E | *Sepp et al., 2001* |
| GH298 | - | +++ | No | No | 3R (99F) | 37294 | E | *Stocker et al., 1997* |
| 796 | *Central complex broad* | +++ | No | No | X |  | E | *Martin-Pena et al., 2006* |

**Supplementary Table 1.** Summary of the Gal4 lines used in the study. Columns specify promoter regions in the Gal4 constructs, presence or absence of expression in the CNS and/or wing disc before 3^rd^ instar larvae (early) or during 3^rd^ instar larvae (L3), insertion sequence location, Blomington Stock Center reference, type of Gal4 constructs (E- enhancer trap with a P-transposase promoter p{GawB}, P- construct including the engogenous promoter of targeted genes p{GaTB} and P-DSCP- Gal4 construct with a core synthetic promoter (DSCP) p{caryP}attP2, and construct generation reference. Dashed lines mean not available information.
